# Four features of temporal patterns characterize similarity among individuals and molecules by glucose ingestion in humans

**DOI:** 10.1038/s41540-022-00213-0

**Published:** 2022-02-08

**Authors:** Suguru Fujita, Yasuaki Karasawa, Masashi Fujii, Ken-ichi Hironaka, Shinsuke Uda, Hiroyuki Kubota, Hiroshi Inoue, Yohei Sumitomo, Akiyoshi Hirayama, Tomoyoshi Soga, Shinya Kuroda

**Affiliations:** 1 Department of Biological Sciences, Graduate School of Science, The University of Tokyo, Bunkyo-ku, Tokyo 113-0033 Japan; 2 Department of Neurosurgery, Graduate School of Medicine, The University of Tokyo, Bunkyo-ku, Tokyo 113-0033 Japan; 3 Molecular Genetics Research Laboratory, Graduate School of Science, University of Tokyo, Bunkyo-ku, Tokyo 113-0033 Japan; 4 Department of Mathematical and Life Sciences, Graduate School of Integrated Sciences for Life, Hiroshima University, Higashi-hiroshima, Hiroshima 739-8526 Japan; 5 Division of Integrated Omics, Research Center for Transomics Medicine, Medical Institute of Bioregulation, Kyushu University, Fukuoka, Fukuoka 812-8582 Japan; 6 Metabolism and Nutrition Research Unit, Institute for Frontier Science Initiative, Kanazawa University, Kanazawa, Ishikawa 920-8640 Japan; 7 Institute for Advanced Biosciences, Keio University, Tsuruoka, Yamagata 997-0052 Japan; 8 CREST, Japan Science and Technology Agency, Bunkyo-ku, Tokyo 113-0033 Japan

**Keywords:** Time series, Systems analysis, Physiology

## Abstract

Oral glucose ingestion induces systemic changes of many blood metabolites related not only to glucose, but also other metabolites such as amino acids and lipids through many blood hormones. However, the detailed temporal changes in the concentrations of comprehensive metabolites and hormones over a long time by oral glucose ingestion are uncharacterized. We measured 83 metabolites and 7 hormones in 20 healthy human subjects in response to glucose ingestion. We characterized temporal patterns of blood molecules by four features: (i) the decomposability into “amplitude” and “rate” components, (ii) the similarity of temporal patterns among individuals, (iii) the relation of molecules over time among individuals, and (iv) the similarity of temporal patterns among molecules. Glucose and glucose metabolism-related hormones indicated a rapid increase, and citrulline and lipids, which indicated a rapid decrease, returned to fasting levels faster than amino acids. Compared to glucose metabolism-related molecules and lipids, amino acids showed similar temporal patterns among individuals. The four features of temporal patterns of blood molecules by oral glucose ingestion characterize the differences among individuals and among molecules.

## Introduction

Glucose metabolism is an important metabolic system directly involved in energy production in humans^1^. After oral glucose ingestion, through absorption from the small intestine, blood glucose concentrations increase, which triggers insulin secretion from pancreatic β cells^2^. Insulin enhances the uptake of blood glucose into tissues such as the liver and skeletal muscles and inhibits hepatic glucose release, and blood glucose concentrations then return to basal concentration^2^. Insulin is also involved in the control of many other metabolites, such as amino acids and lipids. Changes in the concentrations of various blood metabolites before and after oral glucose ingestion in healthy human subjects using metabolomics have been reported^3–11^. These studies did not include measurements of hormones other than insulin. In addition, a few studies investigated changes that occurred more than two hours after oral glucose ingestion^12–14^. However, the detailed temporal changes in the concentration of comprehensive metabolites and hormones over long periods after oral glucose ingestion have yet to be examined.

Insulin secretion accompanies C-peptide secretion, which is a peptide that is cleaved from an insulin precursor (proinsulin) to produce insulin and secreted at an equimolar ratio with insulin^15,16^. C-peptide is used as a clinical marker that reflects insulin secretion because insulin, not C-peptide, is extracted by the liver. Insulin secretion is regulated by incretins, which are hormones secreted from the gastrointestinal tract upon food ingestion; these hormones act on pancreatic β cells to promote insulin secretion^17^. After oral glucose ingestion, gastric inhibitory polypeptide (GIP) and glucagon-like peptide-1 (GLP-1), which are types of incretins, are secreted from the digestive tract, and insulin secretion occurs simultaneously to increase blood glucose concentration. Hormones other than insulin, such as GIP, mutually cross-talk and cooperatively regulate systemic glucose metabolism, and measuring these hormones simultaneously together with blood metabolites are critical for understanding the mechanism of systemic glucose metabolism. However, the differences in the temporal patterns of hormones by oral glucose ingestion among individuals and among molecules have not been compared with those of blood metabolites because hormones and many metabolites have not thus far been simultaneously and comprehensively measured.

The metabolic control by insulin involves the metabolism of various metabolites, including amino acids, lipids (such as free fatty acids), and total ketone bodies, which are controlled by inter-organ communication through the blood after glucose ingestion. For example, in skeletal muscles, proteins are broken down to produce amino acids, which are released into the blood during the fasting state, whereas amino-acid uptake and protein synthesis occur after glucose ingestion^18,19^. In adipose tissue, free fatty acids are released during the fasting state, whereas degradation of triglyceride is inhibited after glucose ingestion and free fatty acids are taken up from blood^5,20^. In the liver, amino acids and free fatty acids are taken up from blood during the fasting state and used as an component for gluconeogenesis and synthesis of ketone bodies^18,21^, whereas the synthesis of ketone bodies is suppressed after glucose ingestion^5,22^. Also, an increase of bile acid and a decrease of urea-cycle-related molecules such as citrulline and ornithine after glucose ingestion have been reported^5,6,11^. Differences in the temporal patterns of metabolites among healthy individuals have been reported^3^. However, the number of molecules was limited to only several types (glucose, insulin, C-peptide, free fatty acids) to study the differences in temporal patterns of molecules among individuals^3^. The response to glucose ingestion of glucose and insulin in type 2 diabetic^7,23–25^ and obese^4^ patients is greater than that of healthy individuals, whereas the response of amino acids and free fatty acids in type 2 diabetic^7^ and obese^4,8^ patients is smaller than that of healthy individuals. However, (1) the differences in the temporal patterns of blood metabolites and hormones by oral glucose ingestion among individuals and among molecules and (2) the characteristics of the temporal patterns have yet to be clarified.

We measured as 601 metabolites and 7 hormones as possible in 20 healthy human subjects in response to glucose ingestion and targeted 76 metabolites and 7 hormones as a result of data preprocessing. We characterized the temporal patterns of molecules among individuals and among molecules by four features: (1) the decomposability into “amplitude” and “rate” components, (2) the similarity of temporal patterns among individuals, (3) the relation of molecules among individuals’ relation over time, and (4) the similarity of temporal patterns among molecules. For the first feature, we classified the temporal patterns of 83 blood molecules into clusters with distinct temporal features (increase or decrease and transient or sustained) and decomposed features of the temporal patterns into “amplitude” and “rate” by principal component analysis. Glucose metabolism-related molecules showed a large amplitude and rapid increase, amino acids showed a large amplitude and slow decrease, and free fatty acids and citrulline showed a larger amplitude and more rapid decrease than amino acids. The amplitude component of the lipids reflected that of glucose, whereas the amplitude component of the amino acids reflected that of insulin. No molecules reflected the rate component of glucose, whereas the rate component of the lipids reflected that of insulin. For the second feature, we quantified the similarity of temporal patterns among individuals. Amino acids and glucose metabolism-related molecules showed similar temporal patterns among individuals. For the third feature, we also quantified the relationship of molecules among individuals’ relation over time. Amino acids showed the constant relation between individuals at each time point over time, whereas glucose metabolism-related molecules and free fatty acids did not. For the fourth feature, we further quantified the similarity of temporal patterns among molecules. Amino acids, lipids, and glucose metabolism-related molecules showed similarity within each group, but they differed from other groups. The temporal pattern of citrulline was intermediate between amino acids, lipids, and glucose metabolism-related molecules. Thus, we demonstrated that blood metabolites have different features of temporal patterns among individuals and among molecules, reflecting selective regulation and action of each group of metabolites and hormones. The point of this study is to quantify the similarity of temporal patterns among individuals and among molecules for a comprehensive number of molecules. For the similarity of temporal patterns among individuals, an earlier study analysed the similarity of temporal patterns of a limited number of target molecules^3^, and we demonstrated the similarity of temporal patterns of a comprehensive number of molecules. For the similarity of temporal patterns among molecules, an earlier study mainly used average value to calculate the similarity of temporal patterns among molecules, and did not take into account individual differences in temporal patterns among individuals in each molecule. We quantified the similarity of temporal patterns among molecules for a comprehensive number of molecules to extend our discussion to metabolic control among individuals and molecules.

## Results

### Measurement of blood metabolites and blood hormones before and after oral glucose ingestion

**Fig. 1:**
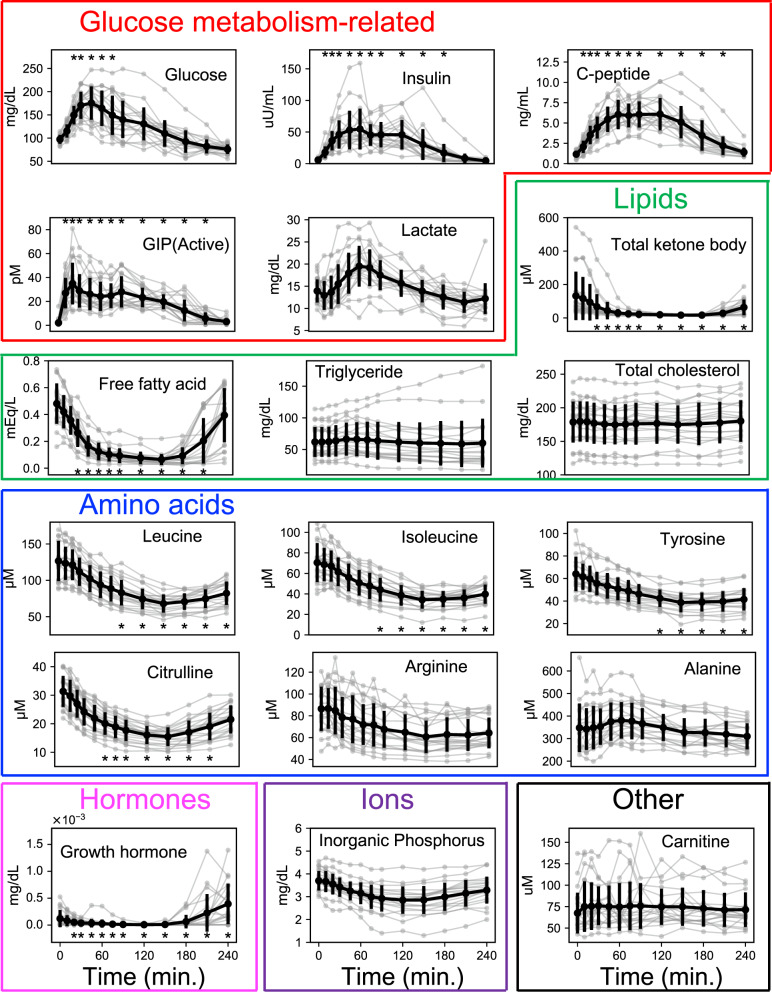
Time courses of blood molecules by glucose ingestion. Time courses of 18 representative blood molecules by glucose ingestion in 20 healthy human subjects. Gray, each subject; black, the mean with a standard deviation of 20 subjects. Red box, glucose metabolism-related molecules; green box, lipids; blue box, amino acids; pink box, hormones; purple box, ions; black box, other metabolites. The asterisks indicate the time points (in minutes, min.) when molecules showed an absolute log2 fold change to the value at fasted state larger than 0.585 (2^0.585^ = 1.5) and a false discovery rate- (FDR-)adjusted *p*-value (*q*-value) <0.1 (Supplementary Fig. 3). Abbreviations for the molecule as follows:GIP (active), gastric inhibitory polypeptide (active).

We obtained blood samples from 20 human healthy subjects at 13-time steps from fasting to 240 min (0, 10, 20, 30, 45, 60, 75, 90, 120, 150, 180, 210, 240 min) after an oral 75 gram (g) glucose ingestion and measured concentrations of 83 molecules (76 metabolites and 7 hormones) (see the “Methods” section, Supplementary Data 1). The timed measurements for 18 representative molecules are shown in Fig. 1 and those of all 83 molecules are shown in Supplementary Fig. 1. Of the 83 molecules, we defined glucose-responsive molecules as the 18 molecules that showed significant changes by oral glucose ingestion. We categorized statistically significant changes into increased and decreased groups (Supplementary Fig. 3). Of the 18 glucose-responsive molecules that changed significantly by glucose ingestion, 6 increased and 12 decreased. The molecules that increased included glucose, insulin, C-peptide, intact-active GIP (active), pyruvate, and total bile acid (Supplementary Fig. 3). The molecules that decreased included cortisol, free fatty acids, total ketone bodies, glutamic acid, citrulline, methionine, isoleucine, leucine, tyrosine, 4-methyl-2-oxopentanoate, growth hormone, and Glu + threo-beta-methylaspartate (Supplementary Fig. 3). We also analyzed the response of the molecules to oral water ingestion as a control, and found that no molecules showed significant changes by oral water ingestion, confirming that the changes we detected reflected a physiological response to glucose ingestion (Supplementary Fig. 2b, Supplementary Data 1).

We selected 18 molecules for each metabolic pathway and showed them in Fig. 1. For the glucose metabolism-related molecules, glucose, insulin, C-peptide, and GIP showed a statistically significant increase by oral glucose ingestion (Fig. 1, Supplementary Figs. 2 and 3). The lipids, free fatty acids, and total ketone bodies (sum of 3-hydroxybutyrate and acetoacetate) significantly decreased, whereas triglycerides and total cholesterol did not show significant changes (Fig. 1, Supplementary Figs. 2 and 3). For the amino acids, leucine, isoleucine, tyrosine, and citrulline significantly decreased, whereas arginine and alanine did not show significant changes (Fig. 1, Supplementary Figs. 2 and 3). For the hormones, the growth hormone significantly decreased (Fig. 1, Supplementary Figs. 2a and 3). Inorganic phosphorus and other metabolites such as carnitine did not show significant changes (Fig. 1, Supplementary Figs. 2 and 3).

For the molecules where an increase was indicated, glucose and insulin changed transiently (Fig. 1). For the molecules where a decrease was indicated, free fatty acids changed transiently, whereas the amino acids changed sustainedly (Fig. 1). A temporal pattern of glucose and leucine was similar among individuals, whereas the temporal pattern of growth hormone largely differed among individuals (Fig. 1). Furthermore, although the temporal patterns of both glucose and leucine were similar among individuals, the relation among individuals of glucose changed over time, whereas that of leucine was constant over time (Fig. 1). For the amino acids, the individual temporal patterns of molecules such as leucine, isoleucine, and citrulline were similar among molecules (Fig. 1) but differed from that of alanine. For the lipids, the temporal pattern of citrulline and free fatty acids were similar (Fig. 1). Taken together, these results indicate that the temporal patterns of the molecules that responded to the glucose ingestion have the following four features: The first feature is that some molecules changed transiently, whereas others changed sustainedly (temporal pattern components). The second feature is that some molecules had a similar temporal pattern among individuals, but others did not (the temporal pattern similarity among individuals). The third feature is that some molecules did not change in the relationships among individuals over time, but others did change (the temporal variation of relation among individuals). The fourth feature is that some molecules had a similar temporal pattern among molecules, but others did not (the temporal pattern similarity among molecules).

### Classification of the temporal patterns of molecules

**Fig. 2:**
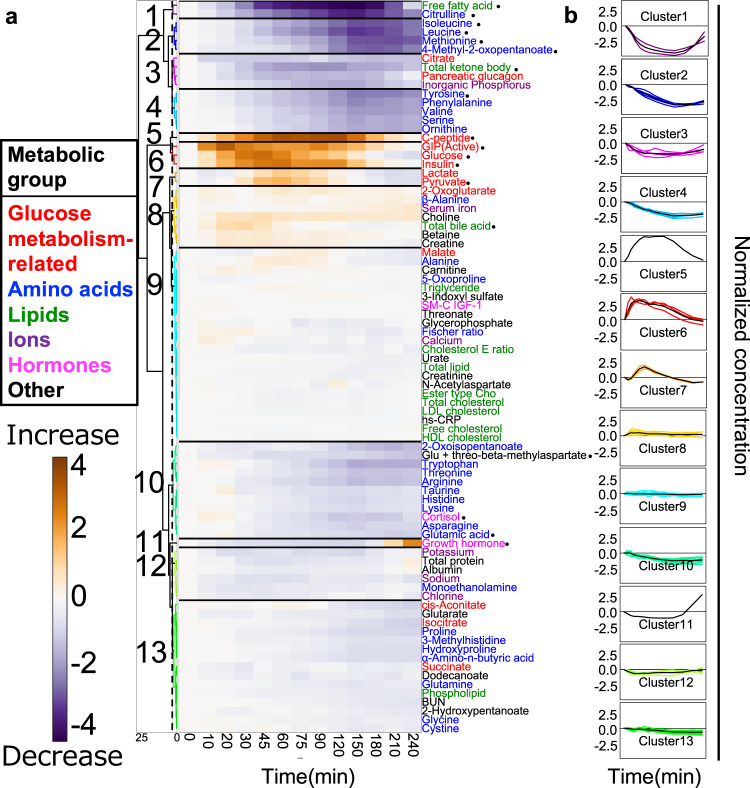
Classification of the temporal patterns of molecules. **a** Heat map of the normalized time course of 83 molecules. Molecules are ordered by hierarchical clustering using Euclidean distance and Ward’s method. The colours and numbers on the tree diagram indicate the cluster that each molecule belongs to. The dashed line indicates the threshold for dividing the cluster. The colours assigned to the names of molecules correspond to the metabolic group (inset). The circles indicate 18 glucose-responsive molecules that showed a significant change by glucose ingestion (Supplementary Fig. 2). **b** Averaged time courses (in minutes (min)) of the molecules for all 13 clusters. The panels show average (thick line) and individual (thin line) time courses of the molecules in a cluster. We performed hierarchical clustering of normalized time courses of 83 molecules. The time courses were divided into 13 clusters characterized by the difference of the temporal pattern of the molecules. We defined the amplitude of the temporal changes of the cluster as large (molecules whose absolute values exceed 2.5, even if just one instance), small (between 1.5 and 2.5), or not clear (<1.5). We also defined transient and sustained patterns of clusters; transient patterns of clusters included molecules that returned to 50% of their maximum values during the time courses; all others were sustained. Note that “Normalized concentration” is dimensionless. Abbreviations for the molecules are as follows: GIP (active) gastric inhibitory polypeptide (active), SM-C IGF-1 somatomedin-C insulin-like growth factor I; ester type Cho ester type cholesterol, HDL cholesterol high-density lipoprotein cholesterol, LDL cholesterol low-density lipoprotein cholesterol, cholesterol E ratio cholesterol ester ratio, BUN blood urea nitrogen, hs-CRP high-sensitivity C-reactive protein. Glu glutamic acid.

We examined the characteristics of the temporal patterns (increase or decrease and transient or sustained) of the molecules by hierarchical clustering of normalized time courses of 83 molecules (Fig. 2, Supplementary Data 2). We normalized time courses as a ratio of relative temporal changes to the fasting values averaged among individuals to the variances among individuals (see the “Methods” section).

The clusters included 18 glucose-responsive molecules (Supplementary Fig. 3). Cluster 1 showed a large transient decrease in citrulline (peaking at 150 min) and free fatty acids (peaking at 150 min). Cluster 2 showed a large, sustained decrease in isoleucine, leucine, methionine, and 4-methyl-2-oxopentanoate. Cluster 3 showed a small transient decrease in total ketone bodies together with the pancreatic glucagon, citrate and inorganic phosphorus. Cluster 4 showed a small, sustained decrease in tyrosine, valine, phenylalanine, and ornithine. For the glucose metabolism-related molecules assigned to cluster 5, 6 showed a large transient increase, including C-peptide (peaking at 120 min), GIP (peaking at 20 min), glucose (peaking at 45 min), and insulin (peaking at 60 min). Cluster 7 showed a small transient increase in pyruvate together with lactate. Cluster 8 showed a small, sustained increase in other metabolites such as choline, betaine, creatine, and total bile acid. Cluster 10 showed a small, sustained decrease in glutamic acid, cortisol, and Glu + threo-beta-methylaspartate. Cluster 11 showed a rapid increase after a sustained decrease in the growth hormone. Clusters 9, 12, and 13 did not show clear temporal patterns including for cholesterol, some amino acids and other metabolites

Both the amino acids and the lipids showed large decreases; the amino acids showed sustained decreases, whereas the lipids showed transient decreases. Although the glucose metabolism-related molecules showed a transient increase, the amplitudes of glucose, insulin, C-peptide, and GIP were larger than those of lactate and pyruvate. Taken together, the temporal patterns of each cluster are characterized by transient or sustained temporal patterns and by their amplitudes.

### Temporal pattern components of molecules

The molecules are characterized by transient or sustained temporal patterns and by their amplitudes, suggesting that the temporal patterns of molecules could be decomposed into a few components. Since clustering analysis alone cannot decompose the differences in temporal patterns into components, we performed principal component analyses of the normalized time courses of 83 molecules to extract the characteristics of the temporal patterns.

**Fig. 3:**
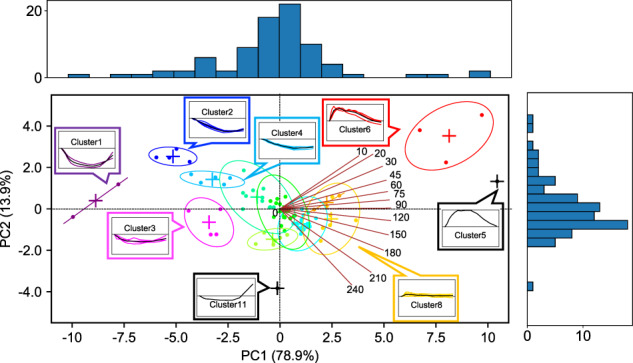
Temporal pattern components of molecules. Biplot of factor loadings and scores of time courses of all molecules. The brown lines indicate factor loading and numbers indicate time points (in minutes (min)). The dots indicate the scores of the molecules. The colours of the dots and ellipses indicate the colours of the clusters classified by hierarchical clustering analysis (Fig. 2). The + symbol indicates the centre coordinates of the ellipses. The panels show average (thick line) and individual (thin line) time courses of the metabolites in a cluster. Numbers in brackets indicate the explained variance rate of each principal component (PC1 and PC2).

The cumulative explained variance rate of the first principal component (PC1) and the second principal component (PC2) (Supplementary Fig. 4A) exceeded 93%, suggesting that the normalized time courses were characterized by two components, PC1 and PC2 (Fig. 3). “Factor loading” is the correlation between the principal component and each variable (time point), and “scores” are the projections of sample points (molecules) on the principal component direction. In short, factor loading represents time dependency in the principal component and scores represent the contributions of each molecule to the principal component.

For the factor loadings, PC1 was large in the positive direction at all time points except for 0 min, and it reached its maximum at 90 min (Fig. 3, red lines; Supplementary Fig. 4b). PC2 changed from a positive to a negative direction over time (Fig. 3, red lines; Supplementary Fig. 4b). According to the time series for the factor loading, PC1 captures the “amplitude and direction” of the temporal pattern, and PC2 captures the “rate” of the temporal pattern (Supplementary Fig. 4c). The transient temporal pattern depends on the ratio of PC1 to PC2. When the signs of PC1 and PC2 are the same (Supplementary Fig. 5, first and third quadrants), the larger the ratio of PC2 to PC1, the more transient the temporal pattern, whereas when the signs of PC1 and PC2 are opposite (Supplementary Fig. 5, second and fourth quadrants), the larger the ratio of PC2 to PC1, the less transient the temporal pattern.

For the scores, the PC1 of clusters 5 and 6 (glucose metabolism-related molecules) was positively high (Fig. 3; Supplementary Fig. 5, first quadrant). The PC1 of cluster 2 (amino acids) was negatively high, and the PC1 of cluster 1 (free fatty acids and citrulline) was negatively higher than the PC1 of other clusters (Fig. 3; Supplementary Fig. 5, second and third quadrants). These results indicate that the molecules in cluster 5 (C-peptide) and cluster 1 had larger positive and negative amplitudes than others, respectively. Cluster 10 (growth hormone) was close to 0 (Fig. 3; Supplementary Fig. 5, third quadrant), indicating that growth hormone showed a small amplitude. For the clusters with a positively high PC1 (a large positive amplitude), the absolute value of the PC2 of cluster 6 (glucose, insulin, and GIP) was higher than that of cluster 5, indicating that the molecules in cluster 6 more rapidly increased than the molecules in cluster 5 (Fig. 3; Supplementary Fig. 5, first quadrant). For the clusters with a negatively high PC1 (a large negative amplitude), the PC2 of cluster 2 (methionine, isoleucine, leucine, and 4-methyl-2-oxopentanoate) was positively higher than the PC2 of clusters 1, indicating that the molecules included in clusters 1 more rapidly decreased than those included in cluster 2 (Fig. 3; Supplementary Fig. 5, second and third quadrants). For cluster 11, the PC2 of growth hormone was negatively high (Fig. 3; Supplementary Fig. 5, third quadrant), indicating rapid growth in growth hormone. The temporal patterns of each cluster indicated by hierarchical clustering analysis can be explained by two components: (1) amplitude and direction and (2) rate. The varying composition of the temporal components of the molecules indicates the possibility of selective metabolic control of each cluster by glucose ingestion.

### Temporal pattern similarity and relation among individuals

**Fig. 4:**
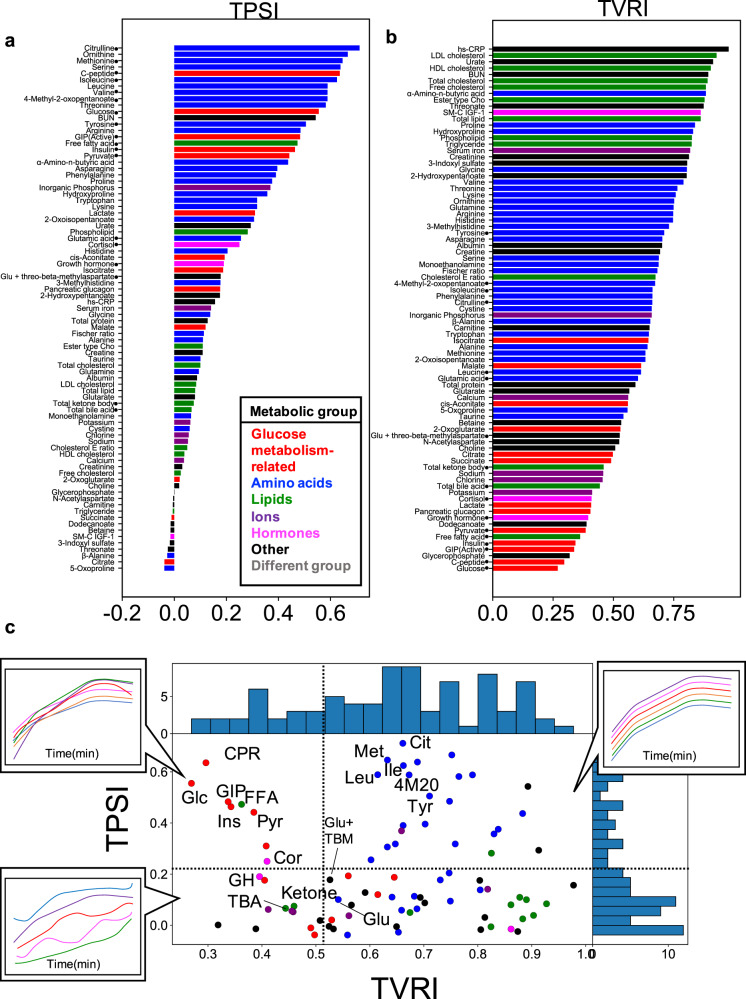
Temporal pattern similarity and relationships among individuals. **a** The temporal pattern similarity among individuals (TPSI) of all 83 molecules. **b** The temporal variation of the relationships among individuals (TVRI) of all 83 molecules. The colour of the bar indicates the metabolic group (inset). The circles indicate 18 glucose-responsive molecules that showed a significant change after glucose ingestion (Supplementary Fig. 3). Abbreviations for the molecules are follows: GIP (active) gastric inhibitory polypeptide (active), SM-C IGF-1 somatomedin-C insulin-like growth factor I, ester type Cho ester type cholesterol, HDL cholesterol high-density lipoprotein cholesterol, LDL cholesterol low-density lipoprotein cholesterol, cholesterol E ratio cholesterol ester ratio, BUN blood urea nitrogen, hs-CRP high-sensitivity C-reactive protein, Glu glutamic acid. **c** The distribution of the TPSI and TVRI values of all molecules. The colours of the dots indicate the metabolic group (inset in (**a**)). The 18 glucose-responsive molecules that showed a significant change after glucose ingestion (Supplementary Fig. 3) are labeled. The time courses (in minutes (min); only trends are shown) are examples where the waveform of each individual changes according to the TPSI and TVRI values. Upper right: Both the TPSI and TVRI values are high. Upper left: The TPSI values are high, but the TVRI values are low. Lower left: Both the TPSI and TVRI values are low. Abbreviations for the molecules that showed a significant change by glucose ingestion are as follows: Cit citrulline, Cor cortisol, CPR C-peptide, FFA free fatty acids, GH growth hormone, GIP gastric inhibitory polypeptide (active), Glc glucose, Glu glutamic acid, Glu + TBM Glu + threo-beta-methylasparate, Ile isoleucine, Ins insulin, Ketone total ketone bodies, Leu leucine, Met methionine, Pyr pyruvate, TBA total bile acid, Tyr tyrosine, 4M2O 4-methyl-2-oxopentanoate.

To examine the temporal pattern similarity among individuals, we defined the temporal pattern similarity among individuals (TPSI) as an index by calculating the correlation coefficient between the time courses connecting all the time courses of the combination of selecting two from all individuals for each molecule (see the “Methods” section). The higher the index number, the more similar the temporal pattern is among individuals. For the 18 glucose-responsive molecules (Supplementary Fig. 3), the molecules whose temporal patterns were similar among individuals were the amino acids (citrulline, methionine, isoleucine, and leucine), and glucose metabolism-related molecules (glucose, insulin, C-peptide, and GIP), and the lipids (free fatty acids) (Fig. 4a). The molecules whose temporal patterns were different among individuals were the hormones (cortisol and growth hormone) and the lipids (total ketone bodies) (Fig. 4a).

We defined TVRI (the temporal variation of the relationships among individuals) as an index of the change of the relationships among individuals over time by calculating an average variation over time of *z*-scored values at each time point for each molecule (see the “Methods” section). The higher the index number, the more constant the relation is among individuals over time. For the 18 glucose-responsive molecules (Supplementary Fig. 3), the relation among the individual amino acids (tyrosine, 4-methyl-2-oxopentanoate, citrulline, and isoleucine) was constant over time. For the glucose metabolism-related molecules (glucose, insulin, and GIP) and the lipids (free fatty acids), the relation among individuals changed over time.

These results indicate the following: (1) The temporal patterns of the amino acids, the glucose metabolism-related molecules, and free fatty acids were similar among individuals (Fig. 4c); and (2) for amino acids, the relation among individuals at each time point was constant over time, whereas for the glucose metabolism-related molecules and free fatty acids, the relation among individuals changed over time (Fig. 4c). These results suggest that the regulation of amino acids is similar and conserved among individuals, whereas the regulation of glucose metabolism-related molecules and free fatty acids are different among individuals.

### Correlation of the values of each molecule between the fasting state and each time point after glucose ingestion

Some of the amino acids such as leucine, isoleucine, tyrosine, citrulline, methionine, and 4-methyl-2-oxopentanoate indicated a higher TVRI value, which may be due to a correlation of values at fasting state with those at each time point. To study that relation, we determined whether the high fasting values in some individuals remained high after oral glucose ingestion. For the 18 glucose-responsive molecules, we correlated the values of each between the fasting state and each time point after glucose ingestion (Supplementary Fig. 3).

**Fig. 5:**
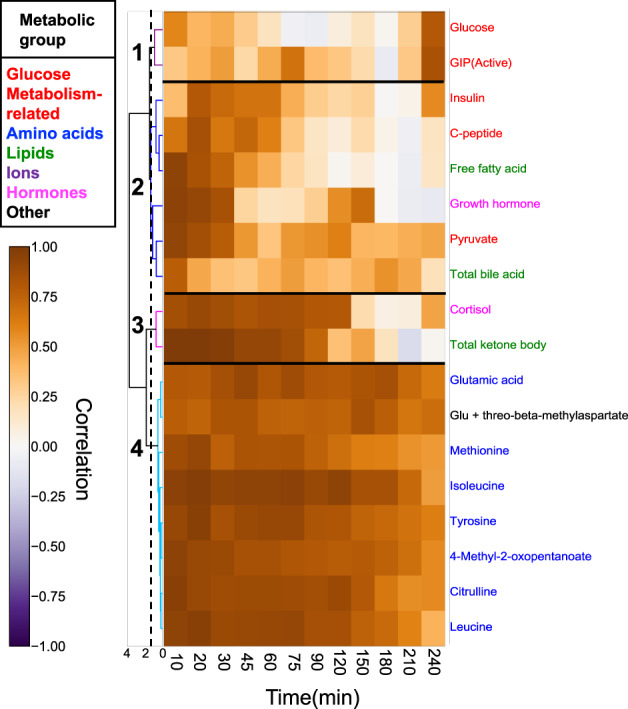
Correlation of values for each molecule. A heat map shows the correlation coefficients between the fasting and each time point (in minutes (min)) among individuals. The 18 glucose-responsive molecules showed a significant change after glucose ingestion was selected. Molecules are ordered by hierarchical clustering using Euclidean distance and Ward’s method. The colours and numbers on the tree diagram indicate the cluster of each molecule. The dashed line indicates the threshold for dividing the cluster. The colours assigned to the names of molecules correspond to the metabolic group (inset). Abbreviations for the molecules are follows: GIP (active), gastric inhibitory polypeptide (active); Glu glutamic acid.

We performed hierarchical clustering of the Pearson’s correlation coefficients of the 18 molecules (Fig. 5). Cluster 1 (the glucose metabolism-related molecules such as glucose and GIP) showed a high correlation only at 240 min after glucose ingestion, but not at other time points (Fig. 5), indicating that the basal blood glucose at fasting state and at 240 min was robustly maintained. Cluster 2 (the other glucose metabolism-related molecules, hormones, and lipids) showed high correlations at the early time points (10–60 min) after glucose ingestion, but the correlations gradually decreased at the later time points (Fig. 5). Cluster 3 (cortisol, total ketone body) showed high correlations for a longer time (10–120 min) than Cluster 2, but the correlations suddenly decreased at the later time points.

Cluster 4 (the amino acids) showed a high correlation at all time points (Fig. 5). The amino acids always maintained a higher correlation among individuals before and after glucose ingestion, indicating that the amino acids had a constant and small variability in relative response to fasting values among individuals. For the glucose metabolism-related molecules, glucose showed a high correlation only at 240 min, whereas insulin showed high correlations at earlier time points; these results indicated that, within each individual, glucose was constantly maintained before and at 240 min after oral glucose ingestion), whereas insulin was constantly maintained at the transient phase (20 min) after oral glucose ingestion. Overall, these results support the idea that the regulation of amino acids is always conserved among individuals.

### The temporal patterns among molecules

The analysis of TPSI revealed that the similarity of temporal patterns among individuals differs depending on the molecule (Fig. 3). The similarities of temporal patterns among blood molecules and among individuals have often separately been analyzed^3,4^. Therefore, we quantified the similarity of temporal patterns among molecules, including information on individual differences in temporal patterns.

To examine the temporal pattern similarity among molecules, we defined TPSM (the temporal pattern similarity among molecules) as an index of the similarity of temporal patterns among molecules by calculating the correlation coefficient between the time courses connecting all individuals (see the “Methods” section). A high positive TPSM value means that molecules are synchronized in the in-phase, whereas the negative TPSM value means that molecules are synchronized in anti-phase.

**Fig. 6:**
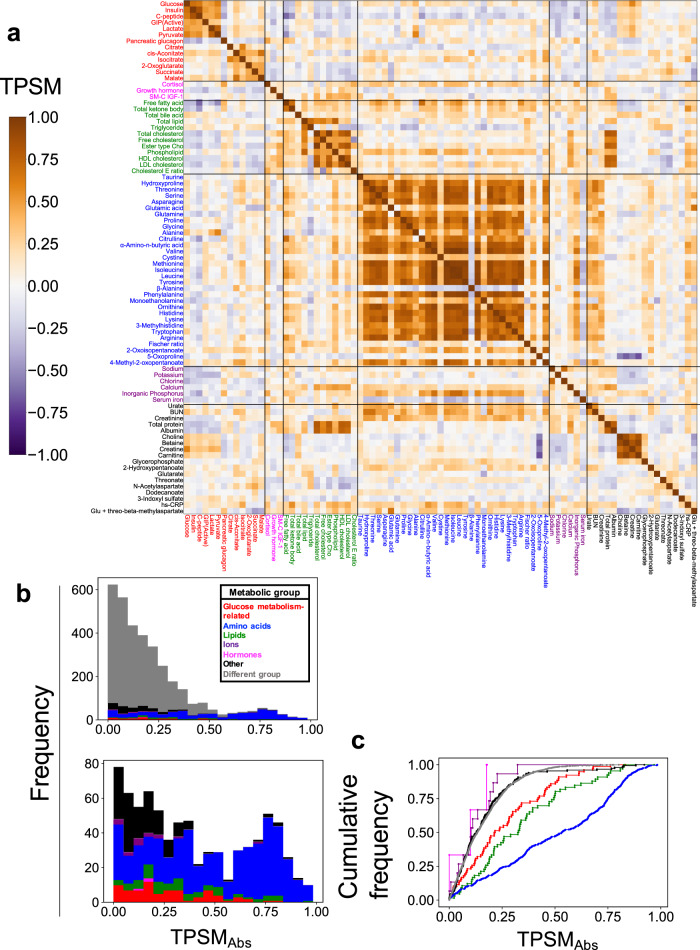
Temporal pattern similarity among blood molecules. **a** Heat map showing the temporal pattern similarity among molecules (TPSM) among all molecules. The molecules are ordered by metabolic group and their label colours correspond to the metabolic group list in the inset in part (**b**). Abbreviations for the molecules are as follows: GIP (active) gastric inhibitory polypeptide (active), SM-C IGF-1 somatomedin-C insulin-like growth factor I, ester type Cho ester type cholesterol, HDL cholesterol high-density lipoprotein cholesterol, LDL cholesterol low-density lipoprotein cholesterol, cholesterol E ratio cholesterol ester ratio, BUN blood urea nitrogen, hs-CRP high-sensitivity C-reactive protein, Glu glutamic acid. **b** The upper histogram shows the distribution of absolute TPSM (TPSM_Abs_) values among all molecules (top). The colours of the bars on the histogram or graph correspond to the metabolic group (inset). The lower histogram shows the distribution of TPSM_Abs_ values among the same metabolic group. **c** The graph shows the cumulative distribution of TPSM_Abs_ among all molecules.

Overall, temporal patterns among molecules in the same metabolic group were similar to those in the different metabolic groups (Fig. 6a). For the glucose metabolism-related molecules (Fig. 6a, red), the temporal patterns of glucose, insulin, C-peptide, and GIP were in-phase, but the temporal pattern of pancreatic glucagon was anti-phase, which is consistent with the counter-action between insulin and glucagon^26^. For the amino acids (Fig. 6a, blue), the temporal patterns of citrulline, methionine, isoleucine, and leucine were in-phase, but the temporal pattern of alanine was anti-phase (Fig. 6a).

To examine the differences in temporal pattern similarity among molecules in the same or different metabolic groups, we colour-coded the distribution of the TPSM values according to its inclusion in the same metabolic group or a different metabolic group (Fig. 6b, upper panel). We defined TPSM_Abs_ as the calculated absolute value of TPSM to eliminate the distinction between positive and negative because we focused on the magnitude of the value. Many of TPSM_Abs_ values within the same group, such as the glucose metabolism-related molecules (shown in red), the amino acids (blue), and the lipids (green) exceeded 0.6 (Fig. 6b, top and bottom), whereas those TPSM_Abs_ values in different metabolic groups were lower than 0.6. Therefore, we set the threshold of the TPSM_Abs_ at 0.6. Because the total number of total molecules in each metabolic group is different, we normalized the TPSM_Abs_ values in the same or different metabolic groups by the total number of total molecules in each group and obtained the ratio of TPSM_Abs_ value for each group (cumulative distribution) (Fig. 6c). According to the shape of this distribution, the different metabolic groups (gray) showed an abrupt increase with an increase in the TPSM_Abs_ value, whereas the glucose metabolism-related molecules (red) and lipids (green) showed gradual increases. The amino acids (blue) showed a more gradual increase, indicating that the group of amino acids showed more similar temporal patterns to each other within the group compared to the other groups.

These results indicated that the temporal patterns among molecules in the same metabolic group were more similar than those in the different metabolic groups. In particular, the amino acids showed the most similar temporal patterns among molecules rather than other groups.

### Connections of molecules exhibiting similar temporal patterns

**Fig. 7:**
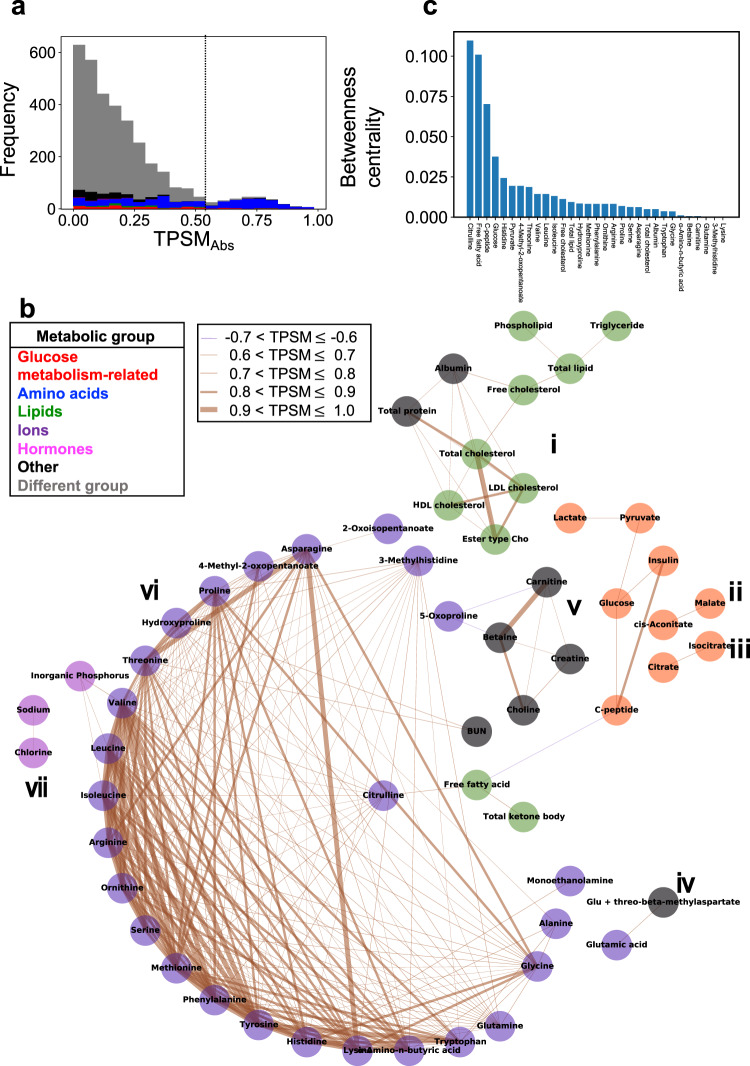
Connections of molecules exhibiting similar temporal patterns. **a** The distribution of absolute temporal pattern similarity (TPSM_Abs_) values among all molecules. The dashed line indicates the threshold of TPSM_Abs_ at 0.6. The colours of the histogram bars correspond to the metabolic group (top left in part **b**). **b** Connections of molecules exhibiting similar temporal patterns. Molecules above the threshold (0.6 in part **a**) are connected. The colours of the molecules correspond to the metabolic group (top left). The colours of the lines indicate positive or negative TPSM values, and the thickness of the lines corresponds to the magnitude of TPSM_Abs_, whereby the thicker the line, the greater the value (top centre). Components (i–vii) are defined as a set of molecules that are not connected to any other molecule. Abbreviations for the molecules are follows: ester type Cho ester type cholesterol, HDL cholesterol high-density lipoprotein cholesterol, LDL cholesterol low-density lipoprotein cholesterol, cholesterol E ratio cholesterol ester ratio, BUN blood urea nitrogen, Glu glutamic acid. **c** Betweenness centrality for the molecules shown in part **b**.

To understand the overall relations among molecules, we created an undirected graph with molecules as nodes and temporal pattern similarities as connections (Fig. 7). We set the threshold of the TPSM_Abs_ at 0.6 (Fig. 7a) and connected molecules above this threshold (Fig. 7b). The connections consisted of seven independent components (Fig. 7b, i–vii). We defined a component as a set of molecules that were not connected to any other molecule (Supplementary Data 3).

The “same” metabolic groups, but not the “different” metabolic groups, were connected, indicating that the molecules in the “same” group showed similar temporal patterns, but the patterns in “different” groups were different. As an example, components ii, iii (for glucose metabolism-related molecules) and component iv (for ions) contained only molecules belonging to the same metabolic group.

The majority of molecules (34 out of 57 molecules)—the glucose metabolism-related molecules (glucose and insulin), amino acids, free fatty acids, and total ketone bodies—were assigned to component v (Fig. 7b). However, the amino acids and glucose metabolism-related molecules were not directly connected. Importantly, citrulline (an amino acid) mediated the connections between other amino acids and glucose metabolism-related molecules through lipids such as free fatty acids and total ketone bodies. This result indicated that citrulline can be a crucial molecule for connecting amino acids to glucose metabolism-related molecules. For the amino acids, only citrulline was connected to the free fatty acids, which was consistent with the classification of citrulline into the same cluster as free fatty acids (Fig. 2).

To quantify the importance of citrulline’s role in connecting amino acids and lipids, we calculated betweenness centrality, an index of the degrees to which a node (molecule) is located on the shortest path between two different nodes^27^. The higher the index, the more connectivity the molecule possesses between nodes. The betweenness centrality of citrulline was 0.1 and was the highest of all molecules, indicating that citrulline indeed serves as a crucial molecule of connecting amino acids and lipids. This result also suggests that the temporal pattern of citrulline is intermediate between amino acids, lipids, and glucose metabolism-related molecules.

### The four features of temporal patterns characterize individual differences among molecules

In previous sections, we characterized the four features of temporal patterns of molecules that respond to glucose ingestion. We characterized the temporal pattern of the molecule as increased or decreased, transient or sustained, and rapid or slow (the first feature). We also characterized the temporal patterns of molecules by the similarity of temporal patterns among individuals (TPSI, the second feature), as the change of the relation among individual magnitude over time (TVRI, the third feature), and as the similarity of temporal patterns among molecules (TPSM, the fourth feature). We will discuss the first feature later. We distinguished the molecules using the values for TPSI, TVRI, and TPSM. Because TPSM is the index defined for pairs of molecules, we defined a quantitative value of TPSM for each molecule, deg_Normalized_, by counting the degrees (deg) in the graph between molecules with a TPSM value above 0.6. The larger the value of deg_Normalized_, the more connections.

**Fig. 8:**
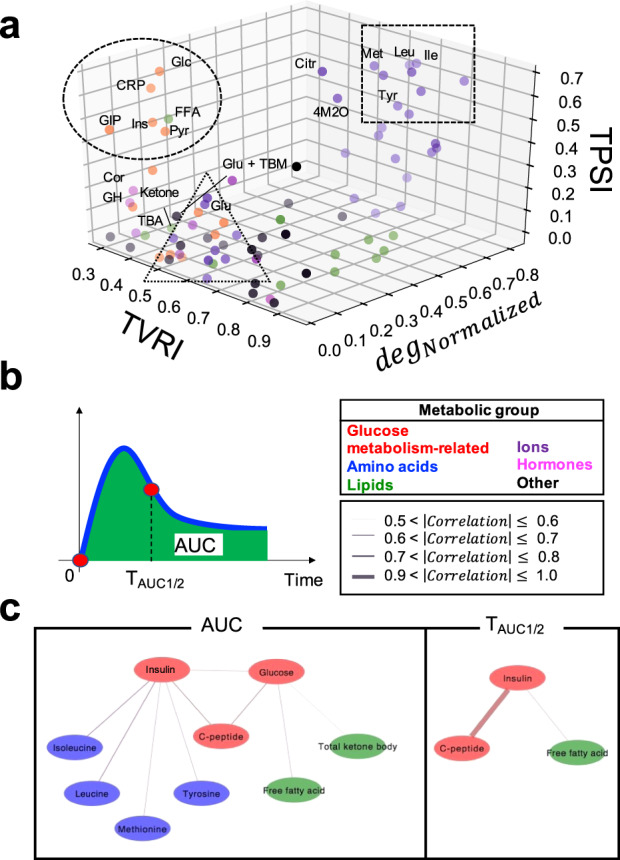
The Four Features of Temporal Patterns That Characterize Individual Differences Among Molecules. **a** The distribution of TPSI, TVRI, and deg_Normalized_ of all molecules. The colours of the dots correspond to the metabolic group (inset). The names of the 18 glucose-responsive molecules that showed a significant change after glucose ingestion (Supplementary Fig. 3) are labelled and their abbreviations are as follows: Cit citrulline, Cor cortisol, CRP C-peptide, FFA free fatty acids, GH growth hormone; Glu + TBM Glu + threo-beta-methylasparate, GIP gastric inhibitory polypeptide (active), Glc glucose, Glu glutamic acid, Ile isoleucine, Ins insulin, Ketone total ketone bodies, Leu leucine, Met methionine, Pyr pyruvate, TBA total bile acid, Tyr tyrosine, 4M2O 4-methyl-2-oxopentanoate. **b** Properties of temporal patterns. AUC is the area under the curve, *T*_AUC1/2_ is the time to reach half of AUC. **c** Connections of molecules showing significant correlation (*q* < 0.1). The colours of the molecules correspond to the metabolic group (middle right). The colours of the lines indicate a positive or negative correlation coefficient, and the thickness of lines corresponds to the magnitude of the correlation coefficient (middle right). Note that |correlation| is an absolute value of the correlation coefficient. The thicker the line, the greater the |correlation|. No pair of the molecules had a 0.8 < |correlation| ≤ 0.9.

We plotted the three indices (TPSI, TVRI, and deg^Normalized^) on a scatter plot (Fig. 8). Because the total number of molecules in each metabolic group differed, we normalized the deg_Normalized_ values in the same metabolic groups by the total number of molecules in each group.

The amino acids with high TPSI and TVRI values also had high deg_Normalized_ values (Fig. 8; dotted square). The glucose metabolism-related molecules and free fatty acids with high TPSI values but low TVRI values had low deg_Normalized_ values (Fig. 8; dotted circle). Taken together, the amino acids whose temporal patterns were similar among individuals and whose relation among individuals was constant over time showed temporal patterns that were similar. The glucose metabolism-related molecules and free fatty acids, whose temporal patterns were similar among individuals but whose relation among individuals frequently changed over time showed temporal patterns that were not similar. Also, it is noteworthy that all the essential amino acids had high TPSI and deg_Normalized_ values (Fig. 8; dotted square), whereas the other amino acids had low TPSI and deg_Normalized_ values (Fig. 8; dotted triangle). These results suggest that essential amino acids show similar temporal patterns among individuals and among essential amino acids, whereas other amino acids do not show the similar temporal patterns among individuals and among the amino acids.

For the first feature, hierarchical clustering analysis showed distinct temporal patterns, and principal component analysis decomposed properties of the temporal patterns into “amplitude” and “rate” (Figs. 2 and 3). The different composition of temporal components of molecules indicated that there may be a selective metabolic control mechanism for each cluster by glucose ingestion.

After glucose ingestion, the “amplitude” and “rate” of the metabolism of amino acids and lipids was controlled by glucose and insulin. We examined the relation between the “amplitude” and “rate” of glucose and insulin and the “amplitude” and “rate” of other molecules. Here, we targeted only glucose-responsive molecules classified in cluster 1–6 (Fig. 2). The scores of principal component analysis captured the features of the ‘averaged’ temporal patterns of each molecule. We needed to calculate the feature values corresponding to the scores for each individual to examine the relationship between the “amplitude” and “rate” of glucose and insulin and the “amplitude” and “rate” of other molecules. We defined the amplitude of the temporal pattern of the molecule as the area under the curve (AUC) (Fig. 8b), because AUC was correlated with the score of PC1 of each molecule (Supplementary Fig. 9A). We also defined the rate of the response of the temporal pattern of the molecules as *T*_AUC1/2_ (the time to reach half of AUC) because *T*_AUC1/2_ was correlated with the ratio of the score of PC1 to PC2 of each molecule (Supplementary Fig. 9B). AUC and *T*_AUC1/2_ are simple and useful because it can be directly calculated from experimental data, and are an indices that can be used for other different experimental data. The fact that AUC and *T*_AUC1/2_ were correlated with PC1 and the ratio of the score of PC1 to PC2, respectively (Supplementary Fig. 9), supports the idea that PC1 and PC2 captured amplitude and rate, respectively.

We calculated the correlation coefficient (*r*) of individual AUC and *T*_AUC1/2_ between glucose, insulin, and other molecules and −log_10_ false discovery related- (FDR-) adjusted *p* value (*q* value). The *q* values were calculated by Storey’s procedure^28^. Note that the transient or sustained and the rapid or slow temporal pattern depends on the ratio of the score of PC1 to the score of PC2 (Fig. 3).

For the AUC, glucose was significantly (*q* < 0.1) correlated with C-peptide (*r* = 0.74), free fatty acids (*r* = −0.61), and total ketone bodies (*r* = –0.54), and insulin was significantly correlated with C-peptide (*r* = 0.71) and amino acids such as isoleucine (*r* = –0.73), leucine (*r* = –0.73), methionine (*r* = –0.65), and tyrosine (*r* = –0.63). These results indicate that glucose has an amplitude component that is similar to that of C-peptide, free fatty acids, and total ketone bodies, whereas insulin has an amplitude component similar to that of C-peptide and amino acids. For *T*_AUC1/2_, glucose was not significantly correlated with any molecule, and insulin was significantly correlated with C-peptide and free fatty acids (*r* = 0.93 and *r* = 0.65, respectively). These results indicate that only insulin has a rate component similar to that of C-peptide and free fatty acids. The correlation of the amplitude between glucose and insulin is consistent with an earlier study^6^. Similarly, the correlation between the amplitude of the insulin and C-peptide is consistent with the result in an earlier study^29^. In addition, a correlation between the rate of C-peptide and insulin was appropriate from the viewpoint of their metabolism.

Taken together, these results indicate that, in the amplitude component, the lipids reflect glucose, whereas the amino acids reflect insulin. In the rate component, no molecules reflect glucose, whereas lipids reflect insulin.

## Discussion

In this study, we demonstrated that blood molecules have different temporal patterns among individuals and among molecules by measuring 76 blood metabolites and 7 blood hormones after oral glucose ingestion in 20 human subjects over 13 time steps. Of the 83 molecules, 18 glucose-responsive molecules showed significant changes before and after glucose ingestion. For the glucose metabolism-related molecules, glucose, insulin, C-peptide, and GIP showed a statistically significant increase (Fig. 1, Supplementary Figs. 2 and 3). For the lipids, free fatty acids and total ketone bodies significantly decreased (Fig. 1, Supplementary Figs. 2 and 3). For the amino acids, leucine, isoleucine, tyrosine, and citrulline significantly decreased (Fig. 1, Supplementary Figs. 2 and 3). For the 18 glucose-responsive, the results were consistent with earlier observations^3–11,30,31^.

We further classified the temporal patterns by hierarchical clustering analysis and decomposed the properties of the temporal patterns into “amplitude” and “rate” by principal component analysis (Figs. 2 and 3). Amino acids including leucine and isoleucine indicated a slow decrease, while free fatty acids and total ketone bodies indicated a rapid decrease (Figs. 2 and 3), both of which are consistent with an earlier study^5^. This result suggested that the suppression of the degradation of triglycerides and the suppression of ketogenesis occur more rapidly than the suppression of proteolysis in response to oral glucose ingestion^5^. In this study, citrulline showed a greater and more rapid decrease than other amino acids, which was similar to the response of free fatty acids. (Figs. 2 and 3), suggesting that the reduction of urea synthesis is also more rapid than the suppression of proteolysis. Citrulline and lipids returned to fasting levels faster than amino acids. This result suggests that the duration of the suppression of the degradation of triglycerides and the suppression of ketogenesis and the reduction of urea synthesis are shorter than that of the suppression of proteolysis. In this study, long time measurements of comprehensive molecules enabled us to discuss the duration of metabolic control.

Glucose metabolism-related molecules (glucose, insulin, C-peptide, and GIP) were classified into clusters 5 and 6, which showed a large increase (Figs. 2 and 3). The temporal patterns of glucose and insulin reached the peak 30 to 60 min after glucose ingestion (Figs. 2 and 3). These results were consistent with earlier observations^5,6,32–34^. C-peptide gradually increased and reached a peak at about 60 min, which was also similar to an earlier study^34^. C-peptide could show a larger increase than insulin because C-peptide was not extracted by the liver^15,16^. GIP reached the peak faster than glucose and insulin (Figs. 2 and 3). This is reasonable because GIP is secreted from the digestive tract with food ingestion and triggers insulin release and subsequent glucose absorption, which are consistent with earlier observation^17,33^.

We quantified the similarity of temporal patterns among individuals by defining TPSI and TVRI. An earlier study classified the temporal patterns of each of the limited molecules according to individual differences, and compared and discussed age, gender, BMI, and glucose tolerance indices among the classified groups^3^. However, comprehensive analysis among molecules and among individuals have not been conducted. In this study, we defined TPSI and TVRI for each molecule, and compared across comprehensive molecules to extend our discussion to individual differences in metabolic control. The temporal patterns of amino acids, glucose metabolism-related molecules, and lipids (free fatty acids) were similar among individuals (Fig. 4). For amino acids, the relations among individuals were constant at each time point, whereas those for the glucose metabolism-related molecules and the free fatty acids were different. This result suggested that the metabolic control of amino acids was similarly conserved among individuals, but that of the glucose metabolism-related molecules and free fatty acids differed among individuals, although all individuals were healthy.

Amino acids were always highly correlated with fasting values at each time point after glucose ingestion (Fig. 5), suggesting that amino acids are controlled by relative value, rather than absolute concentration, within each individual. For the glucose metabolism-related molecules, glucose was highly correlated with fasting values only at 240 min, whereas insulin was highly correlated with earlier time points, suggesting that glucose controls concentrations at 240 min and insulin controls the response during the transitional period (20 min) after oral glucose ingestion.

We quantified the similarity of temporal patterns among molecules by defining TPSM. An earlier study mainly used average value to calculate the similarity of temporal patterns among molecules, and did not take into account individual differences in temporal patterns among individuals in each molecule. Our analysis of TPSI revealed that the similarity of temporal patterns among individuals differs depending on the molecule. Therefore, we quantified the similarity of temporal patterns among molecules, including information on individual differences in temporal patterns. The temporal patterns among molecules in the same metabolic group were more similar than those in the different metabolic groups (Fig. 6). In particular, the amino acids showed the most similar temporal patterns among molecules rather than other groups (Fig. 6). This result is consistent with earlier studies, which indicated that the response before and after glucose ingestion was correlated in the same metabolic group, particularly in the amino acid group^6^. In this study, we confirmed that the correlation of detailed time patterns over 4 h also showed the same result.

Among molecules that showed highly similar temporal patterns, the temporal pattern of citrulline was intermediate among the amino acids, lipids, and glucose metabolism-related molecules (Fig. 7). This result suggests that citrulline shows an intermediate response to these different responsive molecular groups, which is reflected by the suppression of proteolysis (amino acid) and the suppression of degradation of triglyceride (free fatty acids); this response is known as insulin action. The decrease in citrulline by glucose ingestion reflects a reduction in urea synthesis^5^. A sensitivity to glucose ingestion might be similar among citrulline and lipids. The similar responses among different metabolic pathways will be studied in the future.

An earlier but similar study performed correlation analysis using calculated features such as AUC among molecules and showed that some amino acids formed correlation clusters depending on whether ingestion was of glucose alone or glucose + protein-hydrolysate^13^. In this study, we focused on the relation between the amplitude and rate of glucose or insulin and the amplitude and rate of the temporal pattern of other molecules and determined the following: For the amplitude component, the lipid reflected glucose and the amino acid reflected insulin. For the rate component, no molecule reflected glucose and the lipid reflected insulin. After glucose ingestion, the amplitude and rate of the metabolism of amino acid and lipid were controlled by glucose and insulin. For the amplitude component, glucose controlled the lipid, and insulin controlled the amino acid. For the rate component, glucose controlled no molecule, but insulin controls the lipid. The selective reflection of the amplitude and rate components of the lipid and the amino acids with those of glucose and insulin suggested that lipids and amino acids were controlled by the selective temporal components of glucose and insulin.

Temporal patterns may differ depending on demographics of individuals. We conducted linear regressions for difference to fasting value of molecules that changed significantly by glucose ingestion against characteristics of individuals (BMI, Age, Gender) (Supplementary Data 4). Among 18 molecules, only two molecules, GIP and GH, showed significant correlation with gender. Therefore, the temporal patterns of these molecules differ significantly by gender. Other glucose metabolism-related molecules (glucose, insulin, C-peptide), amino acids (leucine, isoleucine, citrulline), and lipids (free fatty acid, total ketone, body) did not show significant correlation with individual characteristics. Therefore, we concluded that the individual differences in the temporal patterns of these molecules were independent of individual characteristics except for GIP and GH.

Main limitations in this study are the sample size and population. However, even in 20 only Japanese subjects, some blood molecule responses were observed after glucose ingestion, and these responses were consistent with previous studies^3–11,33^. The number of men and women were 6 and 14, respectively, in this study, and the similar numbers of men and women would be desirable to draw the conclusions of the effect of gender difference. Further studies are needed to determine whether these findings can be replicated in larger samples. The principal component analyses in this study showed that the temporal patterns of many molecules were decomposed into “amplitude” and “rate”. However, growth hormone showed a rapid increase after a sustained decrease. This means that the temporal pattern of growth hormone cannot be explained by only two components, amplitude and rate. In the future, analysis that can classify such temporal patterns will be necessary.

In conclusion, we quantified the four features of the temporal pattern of molecules that respond to glucose ingestion. The first feature was the decomposability into temporal components. We succeeded in characterizing the temporal patterns of 83 molecules by “amplitude” and “rate.” The second feature was the similarity of temporal patterns among individuals. Amino acids and glucose metabolism-related molecules were similar among individuals. The third feature was the relation among individuals over time. Amino acids did not change the relation among individuals over time, but glucose metabolism-related molecules and free fatty acids did. The fourth feature was the similarity of temporal patterns among molecules. The temporal pattern of citrulline was intermediate between amino acids, lipids, and glucose metabolism-related molecules. The features among individuals and molecules revealed that the amino acids whose temporal patterns were similar among individuals and whose relation among individuals was constant over time showed similar temporal patterns among the amino acids. The glucose metabolism-related molecules and free fatty acids, whose temporal patterns were similar among individuals but whose relation among individuals frequently changed over time did show similar temporal patterns among them. The relationship among the fasting values and the values at each time point after glucose ingestion revealed that amino acids are controlled to maintain the relative value among individuals throughout response to glucose ingestion, insulin is controlled to maintain the concentrations at the transient phase (20 min) after oral glucose ingestion among individuals, and glucose is controlled to maintain the concentrations at 240 min after the response among individuals (Fig. 5).The relation among the amplitude and rate of glucose and insulin and those of other molecules revealed that glucose and insulin controlled amino acid metabolism and lipid metabolism selectively by their temporal components. This study provides new insights into how the metabolism of each molecule among individuals is controlled and what is controlled in the metabolism of each molecule among molecules after glucose ingestion in healthy subjects. The features of the temporal pattern of molecules after glucose ingestion that reflect differences in metabolic control among individuals would be new features for physiological and pathological mechanisms of human systemic glucose metabolism among individuals and for personalized medicine in the future.

## Methods

### Subjects

The study included 20 healthy subjects. The subjects’ profiles are shown in Table 1, and all subjects provided written informed consent.

**Table 1:** Characteristics of subjects.

Characteristics	*N* = 20
Age (years)	29 ± 9 (20–54)
Gender	6 women, 14 men
Height (cm)	168 ± 8 (153–178)
Weight (kg)	59 ± 9 (41–76)
Waist (cm)	74 ± 8 (60–87)
BMI (kg/m^2^)	20.8 ± 2.2 (17.5–25.7)
Fasting glucose (mg/dL)	96 ± 8 (82–115)
2-h glucose (mg/dL)	131 ± 35 (75–238)
Fasting insulin (µU/mL)	5.7 ± 2.1 (1.8–10.8)
2-h insulin (µU/mL)	45.71 ± 23.1 (10.6–95.6)
HOMA-IR	1.4 ± 0.6 (0.5–2.7)
HOMA-β	63 ± 21.5 (24.4–106.6)

Data shows mean ± standard deviation. The values in parentheses indicate the minimum and maximum values. Fasting glucose and fasting insulin indicate fasting values of glucose and insulin, respectively; and 2-h glucose and 2-h insulin indicate values of blood glucose and insulin 2 h after glucose ingestion, respectively.

*2-h* 2-hour, *cm* centimetres, *HOMA-β* homeostasis model assessment of β-cell function, *HOMA-IR* homoeostatic model assessment of insulin resistance, *kg* kilograms, *kg/m*^2^ kilograms per square meter, *mg/dL* milligrams per decilitre, *µIU/ml* million international units per millilitre, *N* total sample size.

### Blood sampling experiment

After 10 h overnight fast, subjects underwent oral glucose tolerance test (OGTT) in the morning. An intravenous catheter was inserted into vein of the forearm and fasting samples were drawn twice, and then a glucose solution containing 75 g glucose (TRELAN-G75 (AJINOMOTO)) or the same amount of water was orally ingested within a few minutes. Blood samples were obtained at 10, 20, 30, 45, 60, 75, 90, 120, 150, 180, 210, 240 min after ingestion as described previously^35^. Subjects remained at rest throughout the test. Blood samples were rapidly centrifuged.

### Sample preparation and measurement

Plasma (40 microliters [μL]) was extracted with the addition of 400 μL of ice-cold methanol containing the internal standards (10 millimolars [mM] l-methionine sulfone [Wako], 100 mM 2-morpholinoethanesulfonic acid [Dojindo], 100 mM D-10-camphorsulfonic acid [Wako]), 400 μL of chloroform, and 120 μL of water. After centrifugation at 10,000 × *g* for 3 min at 4 °C, the separated aqueous layer was filtered through a 5 kilodalton (kDa) cutoff filter (Millipore) to remove protein contamination. The filtrate (300 μL) was lyophilized and dissolved in 20 μL water containing the 2 types of reference compounds (200 μM each of trimesate [Wako] and 3-aminopyrrolidine [Sigma-Aldrich]) for migration time and then injected into the capillary electrophoresis time-of-flight mass spectrometry (CE-TOFMS) system (Agilent Technologies)^36–38^. Among the measured molecules, GIP (active) was measured using an ELISA kit. Blood hormones and some metabolites were measured according to methods developed by LSI Medience Co., Ltd. The methods used to measure each of these molecules are listed in Supplementary Data 5; among these, the amino acid fractions measured by liquid chromatography–mass spectrometry (LC–MS) are listed in Supplementary Data 6, and the metabolites measured by CE-TOFMS are listed in Supplementary Data 7.

### Ethics Committee certification

We complied with Japan’s Ethical Guidelines for Epidemiological Research, and the study as approved by the Institutional Review Board and the Ethics Committee of Tokyo University Hospital. (10264-(4)). Subjects were recruited by the snow-ball sampling.

### Exclusion of blood molecules with a large percentage of missing values

We calculated missing points for each blood molecule by using the time series of all subjects. We excluded the molecules from an analysis target whose percentage of missing points exceeded the top 5% (all 20 subjects × 14 time points × 5% = 14 time points). Supplementary Data 8 shows the percentage of missing points of 25 molecules, including at least one or more missing points among the 83 molecules as an analysis target. In this study, we considered the mean value to be −10, and the fasting value to be 0 min.

### Classification of blood molecules

We classified the 83 blood molecules selected as analysis targets into glucose metabolism-related molecules, lipids, amino acids, ions, hormones, and other metabolites (Supplementary Fig. 2A, Supplementary Data 9). In this study, the classification and colour coding of each molecule is found in Supplementary Fig. 2A.

### The temporal variation of relation among individuals

We defined the temporal variation of relation among individuals (TVRI) as an index of the change of the relation among individuals over time by calculating an average variation over time of *z*-scored values at each time point.

1 $$\bar x_{k,t} = \frac{{\mathop {\sum }\nolimits_{j = 1}^{N_{{\rm {individual}}}} x_{j,k,t}}}{{N_{{\rm {individual}}}}},\quad S_{k,t}^x = \sqrt {\frac{{\mathop {\sum }\nolimits_{j = 1}^{N_{{\rm {individual}}}} (x_{j,k,t} - \bar x_{k,t})^2}}{{N_{{\rm {individual}}} - 1}},}$$

2 $$z_{j,k,t} = \frac{{x_{j,k,t} - \overline x _{k,t}}}{{S_{k,t}^x}},\quad \bar z_{j,k} = \frac{{\mathop {\sum }\nolimits_{t = 0}^{N_{{\rm {time}}}} z_{j,k,t}}}{{N_{{\rm {time}}}}},$$

3 $$S_{j,k}^z = \sqrt {\frac{{\mathop {\sum }\nolimits_{t = 0}^{N_{{\rm {time}}}} (z_{j,k,t} - \bar z_{j,k})^2}}{{N_{{\rm {time}}} - 1}},}$$

4 $$\overline {S_{j,k}^z} = \frac{{\mathop {\sum }\nolimits_{j = 1}^{N_{{\rm {individual}}}} S_{j,k}^z}}{{N_{{\rm {individual}}}}},\;{\rm {TVRI}}_k = 1 - \overline {S_{j,k}^z} .$$

where *x*_*j,k,t*_ indicates the concentration of individual *j* for blood molecule *k* at time point *t*, *N*_individual_ indicates the number of individuals, and *N*_time_ indicates the number of time points. $\bar x_{k,t}$ is the individual mean of *x*_*j,k,t*_ of each molecule and each time point, and $S_{k,t}^x$ indicates the standard deviation (Eq. (1). *z*_*j,k,t*_ is a value obtained by normalizing *x*_*j,k,t*_ by mean centering and variance scaling, and $\bar z_{j,k}$ is the mean of *z*_*j,k,t*_ in the time direction (Eq. (2)). $\bar z_{j,k}$ indicates the time-mean of the relative concentration temporal changes among individuals. Similarly, $S_{j,k}^z$ indicates the standard deviation of *z*_*j,k,t*_ in the time direction (Eq. (3)), which is a measure of how much the relation of individual *j* of molecule *k* changes. We defined TVRI_*k*_ (the relation among individuals over time) by subtracting $\overline {S_{j,k}^z}$ from 1, which is the mean of $S_{j,k}^z$ for all individuals (Eq. (4)). Thus, TVRI_*k*_ indicates how the relation of individual *j* of molecule *k* is constant over time. The higher the value for TVRI_*k*_, the more constant the relation among individuals of molecule *k* over time. High-sensitivity C-reactive protein (hs-CRP) was excluded from this analysis because data about this molecule for the 20 individuals were not available for all time points.

### Normalization of time series

For each blood molecule at each time point after glucose ingestion, we defined a normalized concentration, which is the magnitude of concentration changes averaged among individuals as follows. We also normalized time courses as a ratio of relative temporal changes to the fasting values to the variances among individuals as follows.

5 $$\bar x_{k,t} = \frac{{\mathop {\sum }\nolimits_{j = 1}^{N_{{\rm {individual}}}} x_{j,k,t}}}{{N_{{\rm {individual}}}}},$$

6 $$S_{k,t} = \sqrt {\frac{{\mathop {\sum }\nolimits_{j = 1}^{N_{{\rm {individual}}}} (x_{j,k,t} - \bar x_{k,t})^2}}{{N_{{\rm {individual}}}}},}$$

7 $$\bar S_k = \frac{{\mathop {\sum }\nolimits_{t = 0}^{N_{{\rm {time}}}} S_{k,t}}}{{N_{{\rm {time}}}}},$$

8 $$Y_{j,k,t} = \frac{{x_{j,k,t} - x_{j,k,0}}}{{\bar S_k}},$$

9 $$\bar Y_{k,t} = \frac{{\mathop {\sum }\nolimits_{j = 1}^{N_{{\rm {individual}}}} Y_{j,k,t}}}{{N_{{\rm {individual}}}}}.$$

where *x*_*j,k,t*_ indicates the concentration of the individual *j*, molecule *k*, time point *t*, $\bar x_{k,t}$ indicates the mean of *x*_*j,k,t*_ for all individuals (Eq. (5)), and *S*_*k,t*_ indicates the standard deviation for all subjects (Eq. (6)). $\bar S_k$ indicates the time-mean of *S*_*k,t*_ (Eq. (7)). We normalized *x*_*j,k,t*_ subtracted from *x*_*j,k*,0_ by $\bar S_k$ as *Y*_*j,k,t*_ (Eq. (8)), and calculated the mean of *Y*_*j,k,t*_ as $\bar Y_{k,t}$ (Eq. (9)). The normalized values indicate the ratio of relative temporal changes to the fasting values to the variances among individuals. The greater the temporal changes, or the smaller the variances among individuals, the greater the normalized values.

### The temporal pattern similarity among individuals

We defined the temporal pattern similarity among individuals (TPSI) as an index of the similarity of temporal patterns among individuals as follows.

10 $${{{\boldsymbol{x}}}}_{{{{\boldsymbol{j}}}},{{{\boldsymbol{k}}}}} = \left[ {x_{j,k,0}^\prime , \cdots ,x_{j,k,240}^\prime } \right]$$

11 $${{{\boldsymbol{X}}}} = \left[ {{{{\boldsymbol{x}}}}_{1,{{{\boldsymbol{k}}}}},{{{\boldsymbol{x}}}}_{1,{{{\boldsymbol{k}}}}}, \cdots ,{{{\boldsymbol{x}}}}_{1,{{{\boldsymbol{k}}}}},{{{\boldsymbol{x}}}}_{2,{{{\boldsymbol{k}}}}}, \cdots ,{{{\boldsymbol{x}}}}_{2,{{{\boldsymbol{k}}}}}, \cdots ,{{{\boldsymbol{x}}}}_{{{{\boldsymbol{j}}}},{{{\boldsymbol{k}}}}}, \cdots ,{{{\boldsymbol{x}}}}_{{{{\boldsymbol{j}}}},{{{\boldsymbol{k}}}}}, \cdots ,{{{\boldsymbol{x}}}}_{19,{{{\boldsymbol{k}}}}}} \right]$$

12 $${{{\boldsymbol{Y}}}} = \left[ {{{{\boldsymbol{x}}}}_{2,{{{\boldsymbol{k}}}}},{{{\boldsymbol{x}}}}_{3,{{{\boldsymbol{k}}}}}, \cdots ,{{{\boldsymbol{x}}}}_{20,{{{\boldsymbol{k}}}}},{{{\boldsymbol{x}}}}_{3,{{{\boldsymbol{k}}}}}, \cdots ,{{{\boldsymbol{x}}}}_{20,{{{\boldsymbol{k}}}}}, \cdots ,{{{\boldsymbol{x}}}}_{{{{\boldsymbol{j}}}} + 1,{{{\boldsymbol{k}}}}}, \cdots ,{{{\boldsymbol{x}}}}_{20,{{{\boldsymbol{k}}}}}, \cdots ,{{{\boldsymbol{x}}}}_{20,{{{\boldsymbol{k}}}}}} \right]$$

13 $${\rm {TPSI}}_k = \rho ({{{\boldsymbol{X}}}},{{{\boldsymbol{Y}}}}).$$

where $x_{j,k,t}^\prime$ indicates the concentration difference from fasting value of the individual *j* of molecule *k* at time point *t*,and ***x***_***j***_,_***k***_ indicates the time series vector of *x*_*j,k,t*_ from 0 to 240 min (Eq. (10)). ***X*** is a vector connecting (20−*j*) of each time series vector ***x***_***j***_,_***k***_ from individual *j*th to 19th, and ***Y*** is a vector connecting 19 time series vectors connecting ***x***_***j***+**1**_,_***k***_ from individual (*j* + 1)th to 20th (Eqs. (11) and (12)). We defined *TPSI*_*k*_ (the temporal pattern similarity among individuals) by using *ρ*(***X***,***Y***), which indicates a Pearson’s correlation coefficient between ***X*** and ***Y*** (Eq. (13)). The TPSI indicates a correlation coefficient between the time courses connecting all the time courses of the combination of selecting two from all individuals (_20_*C*_2_ = (20!/(2!18!)) = 190). The higher this index is, the more similar temporal patterns among individuals are.

### The temporal pattern similarity among molecules

We defined the temporal pattern similarity among molecules (TPSM) as an index of the similarity of temporal patterns among molecules as follows:

14 $${{{\boldsymbol{x}}}}_{{{\boldsymbol{k}}}}^\prime = \left[ {x_{1,k,0}^\prime } \right., \cdots ,x_{1,k,240}^\prime ,x_{2,k,0}^\prime , \cdots ,\left. {x_{j,k,0}^\prime , \cdots ,x_{20,k,240}^\prime } \right]$$

15 $${\rm {TPSM}}_{kl} = \rho ({{{\boldsymbol{x}}}}_{{{\boldsymbol{k}}}}^\prime ,{{{\boldsymbol{x}}}}_{{{\boldsymbol{l}}}}^\prime ),$$

16 $${\rm {TPSM}}_{kl}^{{\rm {Abs}}} = \left| {{\rm {TPSM}}_{kl}} \right|.$$

where $x_{j,k,t}^\prime$ indicates the concentration difference from fasting value of the individual *j* of molecule *k* at time point *t*, and ${{{\boldsymbol{x}}}}_{{{\boldsymbol{k}}}}^\prime$ is the time series vector connecting the $x_{j,k,t}^\prime$ (Eq. (14)). We defined the temporal pattern similarity among molecules *k, l* as TPSM_*kl*_ by using $\rho \left( {{{{\boldsymbol{x}}}}_{{{\boldsymbol{k}}}}^\prime ,{{{\boldsymbol{x}}}}_{{{\boldsymbol{l}}}}^\prime } \right)$, which indicates a Pearson’s correlation coefficient between ${{{\boldsymbol{x}}}}_{{{\boldsymbol{k}}}}^\prime$ and ${{{\boldsymbol{x}}}}_{{{\boldsymbol{l}}}}^\prime$ (Eq. (15)). Thus, ${\rm {TPSM}}_{kl}^{{\rm {Abs}}}$ indicates how similar the temporal pattern of molecules *k*, *l* is. A high negative value of TPSM_*kl*_ indicates that temporal patterns are synchronized in anti-phase. Here, some sets of molecules showed negative correlations, but they are at least about −0.686 (min(TPSM_*kl*_) ≈ –0.686). We defined ${\rm {TPSM}}_{kl}^{{\rm {Abs}}}$ by calculating an absolute value of TPSM to eliminate the distinction between positive and negative because we focused on the magnitude of the value (Eq. (16)). We set the threshold of TPSM_Abs_ at 0.6 because Pearson’s correlation coefficient is judged to be moderately high when it is >0.6^39^.

### Betweenness centrality

We performed the Brandes algorithm to calculate the centrality^40^.

### Degree

In the graph between molecules with TPSM above the threshold, we defined deg_Normalized_ as an index that normalizes the number of edges (degree) connected to the molecule *k* by the metabolic group including molecule *k* as follows:

17 $${{{\rm{deg}}}}_{{{{\rm{Normalized}}}}}\left( {{{k}}} \right) = \mathop {\sum}\nolimits_{{{{m}}} < {{{M}}}} {\frac{{\left| {{{e}}} \right|}}{{\left| {{{m}}} \right|}}}$$

where |*e*| indicates the number of edges connected to the molecule *k*, and |*m*| indicates the number of metabolic groups (Supplementary Data 9) including the molecule *k*. *M* indicates a set of metabolic groups.

### Hierarchical clustering analysis

We performed hierarchical clustering of normalized time courses of 83 molecules (Fig. 2) and the correlation coefficients of 18 glucose-responsive molecules (Fig. 5) using Euclidean distance and Ward’s method. For the normalized time courses of 83 molecules (Fig. 2), based on the clustering tree, we defined 13 clusters of molecules that showed the different temporal patterns of the normalized time course among molecules.

For the correlation coefficients of 18 glucose-responsive molecules (Fig. 5), which were based on the clustering tree, we defined four different clusters of molecules that showed the temporal change of the correlation coefficient among molecules.

### Principal component analysis

We performed a singular value decomposition method^41^ for a principal component analysis and an approximate ellipse.

### Molecules that changed significantly by glucose ingestion

We defined the significant change in the concentration of molecules from a fasting state by glucose ingestion as follows. The fold change of the values at each time point over the fasting values was calculated for each molecule. The significance of the change at each time point was tested by two-tailed paired *t*-test for each metabolite. Molecules that showed an absolute log2 fold change >0.585 (20.585 = 1.5) and a −log_10_ false discovery response- (FDR-) adjusted *p* value (*q* value) <0.1 at any time point compared to the fasting state (0 min) were defined as molecules that changed significantly after glucose ingestion (Supplementary Fig. 3). The *q* values were calculated using Storey’s procedure (Storey, 2002). To define an increase or decrease in time courses with changes in both directions at different times, we used the direction of change compared to a fasting state at the earliest time point that showed a significant change. Molecules that responded to oral water ingestion were determined using the same procedure that defined molecules that changed significantly after glucose ingestion (Supplementary Fig. 3, Supplementary Data 1).

## Supplementary information

Combined SUPPLEMENTAL MATERIAL

Combined SUPPLEMENTAL MATERIAL

Supplementary Data 9

Supplementary Data 8

Supplementary Data 7

Supplementary Data 6

Supplementary Data 5

Supplementary Data 4

Supplementary Data 3

Supplementary Data 2

Supplementary Data 1

Combined SUPPLEMENTAL MATERIAL_MARKED

## Data Availability

All data generated or analysed during this study are included in this published article and its supplementary materials files.

## Code availability

The code files are freely available at http://kurodalab.org/publication/info/fujita2021/.
