# Supplementary material for: Four features of temporal patterns characterize similarity among individuals and molecules by glucose ingestion in humans
Source: NPJ Syst Biol Appl. 2022 Feb 8;8:6. doi: 10.1038/s41540-022-00213-0 (PMC8826934; doi:10.1038/s41540-022-00213-0)
Supplement: Supplementary file 1 — Combined SUPPLEMENTAL MATERIAL [file 41540_2022_213_MOESM1_ESM.docx]

**Supplementary Information for**

**“Four features of temporal patterns characterize similarity among individuals and molecules by glucose ingestion in humans”**

Authors

Suguru Fujita^1,†^, Yasuaki Karasawa^2,†^, Masashi Fujii^1,3,4^, Ken-ichi Hironaka^1^, Shinsuke Uda^5^, Hiroyuki Kubota^5^, Hiroshi Inoue^6^, Yohei Sumitomo^1^, Akiyoshi Hirayama^7^, Tomoyoshi Soga^7^, Shinya Kuroda^1,3,8^ ^*^

Affiliations

^1^Department of Biological Sciences, Graduate School of Science, The University of Tokyo, Tokyo, 113-0033, Japan

^2^Department of Neurosurgery, Graduate School of Medicine, The University of Tokyo, Tokyo, 113-0033, Japan

^3^Molecular Genetics Research Laboratory, Graduate School of Science, University of Tokyo, Bunkyo-ku, Tokyo 113-0033, Japan

^4^Department of Mathematical and Life Sciences, Graduate School of Integrated Sciences for Life, Hiroshima University, Hiroshima, 739-8526, Japan

^5^Division of Integrated Omics, Research Center for Transomics Medicine, Medical Institute of Bioregulation, Kyusyu University, Fukuoka, 812-8582, Japan

^6^Metabolism and Nutrition Research Unit, Institute for Frontier Science Initiative, Kanazawa University, Ishikawa, 920-8640, Japan

^7^Institute for Advanced Biosciences, Keio University, Tsuruoka, Yamagata, 997-0052, Japan

^8^CREST, Japan Science and Technology Agency, Tokyo, 113-0033, Japan

Correspondence: Shinya Kuroda ([skuroda@bs.s.u-tokyo.ac.jp](mailto:skuroda@bs.s.u-tokyo.ac.jp))

^†^These authors contributed equally.

**Corresponding Author:**

*Correspondence: skuroda@bs.s.u-tokyo.ac.jp

# **This file includes Supplementary Figures 1–9.Supplementary Figures**

Supplementary Figure 1 Time courses of all 83 blood molecules in 20 subjects by glucose ingestion

Time courses of all 83 blood molecules by glucose ingestion in 20 healthy human subjects. For each graph, the gray lines represent each subject and the black line is the mean with a standard deviation of 20 subjects. The graphs are grouped in colored boxes as follows: Red box, glucose metabolism-related molecules; green box, lipids; blue box, amino acids; pink box, hormones; purple box, ions; black box, other metabolites. The asterisks indicate the time points when molecules showed an absolute log2 fold change to the value at fasting state greater than 0.585 (2^0.585^ = 1.5) and a false discovery related- (FDR-) adjusted *p* value (*q* value) less than 0.1 (Supplementary Figure 3). Abbreviations for the molecules are follows: GIP (active), gastric inhibitory polypeptide (active), SM-C IGF-1, somatomedin-C insulin-like growth factor I; ester type Cho, ester type cholesterol; HDL cholesterol, high density lipoprotein cholesterol; LDL cholesterol, low density lipoprotein cholesterol; cholesterol E ratio, cholesterol ester ratio; BUN, blood urea nitrogen; hs-CRP, high-sensitivity C-reactive protein; Glu, glutamic acid. Abbreviations for the units are follows: mg/dL, milligrams per deciliter; μU/mL, microunits per milliliter; ng/mL, nanograms per milliliter; pM, pico molar; pg/mL, pico grams per milliliter; μM, micromolar; μg/dL, micrograms per deciliter; mEq/L, milliequivalents per liter; g/dL, grams per deciliter.

Supplementary Figure 2 Classification of molecules

(a) The number and percentage of molecules included in metabolic groups. The colors of the pie sectors indicate metabolic groups. (b) Time courses of glucose and insulin to oral glucose ingestion (red) and oral water ingestion (cyan) in 3 healthy human subjects. The means and SEMs of 3 subjects are shown.

Supplementary Figure 3 The 18 molecules that changed significantly by glucose ingestion

(a) Distribution of mean of log2 fold change of values at each time point divided by the fasting values for each molecule. The dashed line indicates that the absolute log2 fold change is 0.585 (2^0.585^ = 1.5). (b) Volcano plot of log2 fold change and −log10 false discovery related- (FDR-) adjusted *p* value (*q* value). The significance of the change at each time point was tested by two-tailed paired t-test for each molecule. The *q* values were calculated by Storey’s procedure (Storey, 2002). Molecules that showed an absolute log2 fold change larger than 0.585 (2^0.585^ = 1.5) and an FDR-adjusted *p* value (*q* value) less than 0.1 at any time point were defined as molecules that changed significantly after glucose ingestion. The vertical and horizontal dashed lines indicate the absolute value log2 fold change of 0.585 (2^0.585^ = 1.5) and the FDR-adjusted *p* value (*q* value) of 0.1, respectively. The colors of dots indicate the increase of (brown) or decrease of (purple) a molecule at any point. (c) The number and percentage of molecules that increased or decreased and did not change. The colors of the pie sectors indicate the increase of (brown) or decrease of (purple) a molecule at any point. (d) For the molecules that increased or decreased, the colors of the molecule names correspond to the metabolic groups (list at bottom left). The number of time points at which molecules showed a significant change is shown. Of the 83 molecules, 18 significantly changed after glucose ingestion. We categorized those that statistically significantly changed into increased and decreased groups. If molecules showed a change by decreasing followed by an increase at different time points, such as for the free fatty acids, citrulline, and growth hormone, we included them in the decreased group. Of the 18 molecules that changed significantly after glucose ingestion, 6 increased and 12 decreased. Molecules that increased (7.2%) included glucose, insulin, C-peptide, GIP, pyruvate, and total bile acid (Fig.1, Supplementary Figures 1 and 3). Molecules that decreased (14.5%) included free fatty acids, total ketone bodies, amino acids (such as leucine, isoleucine, and citrulline), and growth hormone (Fig.1, Supplementary Figures 1 and 3). We also analyzed blood molecules in healthy humans who were orally given an equivalent amount of water; the blood amino acids and lipids showed no changes (Supplementary Table 1), confirming that the changes we detected reflected a physiological response to the oral glucose ingestion. Abbreviations for the molecules are follows: GIP (active), gastric inhibitory polypeptide (active), Glu, glutamic acid.

Supplementary Figure 4 Principal component analysis of temporal patterns of molecules
(a) The cumulative explained variance rate of the principal components (PC). (b) Heat map showing factor loading. (c) Time courses of factor loadings of PC1 (left) and PC2 (right). The lines indicate the time courses of factor loadings of each molecule. The colors of the lines indicate the clusters (numbered 1 to 13) as shown in the color bar to the right. The numbers in brackets indicate the explained variance rate of each PC. (d) Heat map of scores. Abbreviations for the molecules are follows: SM-C IGF-1, somatomedin-C insulin-like growth factor I; ester type Cho, ester type cholesterol; HDL cholesterol, high density lipoprotein cholesterol; LDL cholesterol, low density lipoprotein cholesterol; cholesterol E ratio, cholesterol ester ratio; BUN, blood urea nitrogen; hs-CRP, high-sensitivity C-reactive protein.

Supplementary Figure 5 Temporal patterns explained by PC1 and PC2

Score plot of time courses of all molecules. The dots indicate the scores of molecules. The colors of the dots correspond to the colors of the clusters classified by hierarchical clustering analysis (Fig. 2). The small panels for 11 of the molecules show time courses of factor loadings explained by PC1 (red dashed line) or PC2 (blue dashed line) and the sum of them (black dashed line). The 18 molecules that showed a significant change after glucose ingestion (Supplementary Figure 3) are labeled. A + or – symbol indicates the sign of each PC. The dashed lines indicate the values that divide the range of PC1 (red) and PC2 (blue) into four equal parts. The placement of the High and Low labels was determined by the absolute value of each PC in positive and negative directions. Unit is shown in the lower right panel (Example) .Note that “Factor loadings” is dimensionless. Abbreviations for the 18 molecules are follows: Cit, citrulline; Cor, cortisol; CRP, C-peptide; FFA, free fatty acids; GH, growth hormone; Glu+TBM, Glu+threo-beta-methylasparate; GIP, gastric inhibitory polypeptide (active); Glc, glucose; Glu, glutamic acid; Ile, isoleucine; Ins, insulin; Ketone, total ketone bodies; Leu, leucine; Met, methionine; Pyr, pyruvate; TBA, total bile acid; Tyr, tyrosine; 4M2O, 4-methyl-2-oxopentanoate.

Supplementary Figure 6 Components and nodes of connections of molecules for multiple thresholds of TPSM

(a) Number of components (blue) and number of nodes (yellow) at the indicated threshold for the absolute value of the temporal pattern similarity among molecules (TPSM_Abs_). We define a component as a set of molecules that were not connected to any other molecule. Connections above the threshold were selected, and the numbers of components and nodes were counted. The dashed line represents the threshold (TPSM_Abs_ = 0.6). (b) The mean of component size (blue) and the variance of component size (yellow) at different TPSM_Abs_ thresholds. The dashed line indicates the threshold (TPSM_Abs_ = 0.6). We examined the change in the mean of component size, the variance of the component size, the number of components, and the number of nodes at different TPSM_Abs_ thresholds. We selected connections above the threshold and counted numbers of components and nodes. We also calculated the mean of component size and the variance of component size. For a TPSM_Abs_ around the threshold, the gradual change in the number of nodes, the mean of component size, and the variance of component decrease, indicating that the relation between components does not change abruptly by changing the threshold of a TPSM_Abs_. However, because the number of components reaches a peak at the threshold of TPSM_Abs_ = 0.65, we also examined the connection of molecules at different TPSM_Abs_ thresholds (Supplementary Figure 7 and 8).

Supplementary Figure 7 Connections of molecules exhibiting similar temporal patterns (threshold; TPSM_Abs_ = 0.55)

(a) The distribution of absolute temporal pattern similarity (TPSM_Abs_) values among all molecules. The dashed line indicates the threshold of TPSM_Abs_ at 0.55. The colors of the histogram bars correspond to the metabolic group (top left in part b). (b) Connections of molecules exhibiting similar temporal patterns. Molecules above the threshold (0.55 in part A) are connected. The colors of the molecules correspond to the metabolic group (top left). The colors of the lines indicate the positive or negative of TPSM values, and the thickness of the lines corresponds to the magnitude of TPSM_Abs_, whereby the thicker the line, the greater the value (top center). Components (i to vi) are defined as a set of molecules that are not connected to any other molecule. Abbreviations for the molecules are follows: ester type Cho, ester type cholesterol; HDL cholesterol, high density lipoprotein cholesterol; LDL cholesterol, low density lipoprotein cholesterol; cholesterol E ratio, cholesterol ester ratio; BUN, blood urea nitrogen; Glu, glutamic acid. (c) Betweenness centrality for the molecules shown in part b. The connections consist of six independent components for the threshold of TPSM_Abs_ at 0.55 (Supplementary Figure 7B, i-vi). For the connection for the threshold of TPSM_Abs_ at 0.55, the majority of the molecules (41 out of 60) such as glucose metabolism-related molecules (glucose and insulin), amino acids, free fatty acids, and total ketone bodies were assigned to component iv. The amino acids and glucose metabolism-related molecules are directly connected through alanine and pyruvate, not through lipids, which is consistent with pyruvate degrading into alanine by glycolysis (Berg JM, et al., 2002). The other amino acids (citrulline, arginine, and leucine), mediated amino acids, and glucose metabolism-related molecules are directly connected through the lipids. The betweenness centrality of the glucose was 0.1 and was the highest of all molecules.

Supplementary Figure 8 Connections of molecules exhibiting similar temporal patterns (threshold TPSM_Abs_ = 0.65)

(a) The distribution of absolute temporal pattern similarity (TPSM^Abs^) values among all molecules. The dashed line indicates the threshold of TPSM_Abs_ at 0.55. The colors of the histogram bars correspond to the metabolic group (top left in part b). (b) Connections of molecules exhibiting similar temporal patterns. Molecules above the threshold (0.65 in A) are connected. Abbreviations for the molecules are follows: ester type Cho, ester type cholesterol; HDL cholesterol, high density lipoprotein cholesterol; LDL cholesterol, low density lipoprotein cholesterol; cholesterol E ratio, cholesterol ester ratio; BUN, blood urea nitrogen; Glu, glutamic acid. (c) Betweenness centrality of the molecules in part b. The connections consist of nine independent components for the threshold of TPSM_Abs_ at 0.65 (Supplementary Figure 8B, i-ix). For the connection for the threshold of TPSM_Abs_ at 0.65, molecules such as amino acids, free fatty acids, and total ketone bodies made up component viii (part b), whereas glucose metabolism-related molecules were not included in this component. This is the reason why the number of components was increased (Supplementary Figure 6). Citrulline mediated the lipids and other amino acids, which indicated that the connection between the lipids and the amino acids through citrulline was stronger than the connection between the glucose metabolism-related molecules and the amino acids through the lipids. Taken together with Supplementary Figure 7, lowering the threshold resulted in the connection of amino acids other than citrulline to lipid and glucose metabolism-related molecules, whereas increasing the threshold resulted in the loss of connection between lipid and glucose metabolism-related molecules. Thus, depending on the threshold, some amino acids showed a temporal pattern similar to lipids and glucose metabolism-related molecules such as free fatty acids and lactate, whereas citrulline was the molecule that showed the temporal pattern most similar to free fatty acids.

Supplementary Figure 9 AUC, T_AUC1/2_, and principal component scores

(a) The distribution of the area under the curve (AUC) of 13 molecules and the score of principal component 1 (PC1). The color of the dots indicates the metabolic group (see inset). In the upper left, *r* and *p* indicate the correlation coefficient and *p*-value, respectively. (b) The distribution of the response of the temporal pattern of the molecule (T_AUC1/2_) of 13 molecules and the ratio of the score of PC1 and principal component 2 (PC2). The color of the dots indicates the metabolic group (see inset in part A). In the upper right, *r* and *p* indicate the correlation coefficient and *p* value, respectively. Note that only molecules with a high response amplitude were targeted (Supplementary Figure 5). (c) The distribution of the coefficient of variation (CV) of AUC and T_AUC1/2_. Molecules are labeled as follows: Cit, citrulline; CRP, C-peptide; FFA, free fatty acids; GIP, gastric inhibitory polypeptide (active); Glc, glucose; Ile, isoleucine; Ins, insulin; Ketone, total ketone bodies; Leu, leucine; Met, methionine; Tyr, tyrosine; 4M2O, 4-methyl-2-oxopentanoate. Amino acids and free fatty acids had low CVs of both AUC and T_AUC1/2_. Glucose and Total ketone bodies had high CVs of both AUC and T_AUC1/2_. Glucose metabolism-related molecules, except glucose, had low CVs of AUC, but high CV of T_AUC1/2_.

Supplementary Data 1 Metabolites and hormones data

Metabolites and hormones data in healthy human before and after oral glucose ingestion.

Supplementary Data 2 Characteristics of temporal patterns of molecules

## Characteristics of temporal patterns of molecules

Supplementary Data 3 Molecules exhibiting the similar temporal patterns

Molecules exhibiting the similar temporal patterns

Supplementary Data 4 Correlation of changed significantly by glucose ingestion with individual characteristics

Correlation of changed significantly by glucose ingestion with individual characteristics

Supplementary Data 5 Measurement methods of some metabolites and hormones

Measurement methods of some metabolites and hormones

Supplementary Data 6 The amino acid fraction measured by LC-MS

The amino acid fraction measured by LC-MS

Supplementary Data 7 Molecules measured by CE-TOFMS

Molecules measured by CE-TOFMS

Supplementary Data 8 The percentage of missing points of 25 molecules including at least one or more missing points

The percentage of missing points of 25 molecules including at least one or more missing points

Supplementary Data 9 Classification of molecules

Classification of molecules
